# Intestinal carriage of methicillin-resistant *Staphylococcus aureus* in nasal MRSA carriers hospitalized in the neonatal intensive care unit

**DOI:** 10.1186/2047-2994-3-14

**Published:** 2014-04-23

**Authors:** Akihiro Nakao, Teruyo Ito, Xiao Han, Yu Jie Lu, Ken Hisata, Atsushi Tsujiwaki, Nobuaki Matsunaga, Mitsutaka Komatsu, Keiichi Hiramatsu, Toshiaki Shimizu

**Affiliations:** 1 Department of Pediatrics, Faculty of Medicine, Juntendo University, 2-1-1 Hongo, Bunkyo-ku, Tokyo 113-8421, Japan; 2 Department of Bacteriology, Faculty of Medicine, Juntendo University, 2-1-1 Hongo, Bunkyo-ku, Tokyo 113-8421, Japan; 3 Department of Infection Control Science, Graduate School of Medicine, Juntendo University, 2-1-1 Hongo, Bunkyo-ku, Tokyo 113-8421, Japan

**Keywords:** MRSA, Infection control, Intestinal carriage, Nosocomial infection, NICU

## Abstract

**Background:**

The current data regarding the correlation between the methicillin-resistant *Staphylococcus aureus* (MRSA) clones carried in the nasal cavity and digestive tract are inadequate.

**Methods:**

MRSA strains were isolated from both the feces and nasal swabs of 21 nasal-MRSA carriers ranging from 10 to 104 days of age treated at the neonatal intensive care units of two hospitals. The molecular epidemiological characteristics of the isolates were determined: multilocus sequence types, *spa*-types, staphylococcal cassette chromosome *mec* (SCC*mec*) types, carriage of four exotoxin genes, and genes contained in commercially available kit.

**Results:**

The feces of all nasal carriers contained MRSA at levels ranging from 4.0 × 10^2^ to 2.8 × 10^8^ colony forming units/g feces. The MRSA clones isolated from the feces and the nasal swabs of each patient were the same. Four MRSA clones, clonal complex (CC) 8-SCC*mec* IVl, CC8-SCC*mec* IVb, CC1-SCC*mec* IVa and CC5-SCC*mec* IIa were identified from 21 patients. All CC8-SCC*mec* IVl strains and one of three CC5-SCC*mec* IIa strains carried the toxic shock syndrome toxin gene.

**Conclusions:**

The feces of tested MRSA carriers contained the same MRSA clones as the nasal isolates in considerable amounts, suggesting that more careful attention should be paid for the handling of excrement in the case of newborn babies or infants than that of adults.

## Introduction

Methicillin-resistant *Staphylococcus aureus* (MRSA) is an important causative pathogen of healthcare-associated infections. It is well known that MRSA strains carried by an infected individuals, asymptomatic carrier or contaminated objects are transmitted via several routes, e.g., direct contact with an infected individual, asymptomatic carrier or contaminated object, the airborne transmission of floating cells, etc. [1]. To control the infection of MRSA, screening of MRSA nasal-carrier is conducted generally at hospitals, since the mucosal membrane of the nasal cavity is a well-known niche for Staphylococcal strains and nasal colonization by MRSA is a well-established risk factor for hospital-acquired MRSA infection among the causes of nosocomial MRSA infection [2-6].

However, MRSA strains colonize at the area other than nasal swabs, and the colonization at those areas was regarded to be another risk factor for MRSA dissemination, too. Universal screening of all hospitalized patients and selective screening limited to high-risk patients, such as those admitted to the intensive care unit or scheduled for surgery, are routinely conducted [7]. About 40% of individuals with nasal colonization are also colonized in other areas, including the throat, perineum and axilla in adults [8,9]. Acton et al. reviewed cases involving the intestinal carriage of *S. aureus*[10]. Ammeriaan et al. reported that one of the causes of treatment failure for MRSA carriers might be due to the presence of strains colonized at extra-nasal sites [11]. Although MRSA infection is decreasing as results of infection control e.g., performing standard precaution, active surveillance culture, and cohorting, outbreak of MRSA strains still occurred. It has been reported that invasive nosocomial infections predominantly occur in children younger than 1 year of age, with an incidence of 14.7 per 100,000, versus 0.3-1.0 per 100,000 in older children [5] and the risk factors associated with an increased rate of infections in the neonatal intensive care unit (NICU) were suggested. These included the presence of invasive devices, exposure to broad-spectrum antibiotic agents, the use of parenteral nutrition, overcrowding and poor staffing ratios [12].

We presumed that the feces of neonates and infants might contain MRSA strains at considerable amount and would have the possibility to serve as a potential source of MRSA dissemination in the NICU if the contact precautions are inconsistent. Furthermore, the reports comparing the characteristics of MRSA strains isolated from the stool and the nasal cavity in MRSA-positive newborn babies and infants are inadequate. We aimed at isolating and characterizing MRSA strains from the feces of MRSA nasal carriers admitted to the NICU. In this study, we estimated the number of MRSA strains in feces and compared the genetic characteristics of strains isolated from nasal swabs and feces.

## Materials and methods

### Subjects

Patients who were judged to be positive for MRSA by nasal screening were selected from among neonate and infant patients admitted to the NICUs between January 2013 and June 2013 at two university-affiliated tertiary hospitals (*J*: Juntendo University Hospital and *S*: Juntendo University Shizuoka Hospital). At the two hospitals, screening for MRSA was performed on every patient at admission, as well as every two weeks after hospitalization. MRSA screening was conducted by inoculating nasal swabs to CHROMagar MRSA (Kanto Chemical Co., Tokyo, Japan), a selective choromogenic agar containing cephamycin [13]. Briefly, one hundred and sixty-nine patients underwent nasal screening, including 100 males and 69 females ranging from 0 to 171 days of age, with an average age of 19.6 days. The average weight was 2,446 g, ranging from 576 to 4,481 g. A total of 26 of the 169 tested patients were found to be MRSA carriers based on the screening specimens. Among the 26 MRSA carriers, 21 patients were selected for this study. Five patients were excluded; two patients were excluded because they had been administered vancomycin intravenously before sample collection for this study, and three patients were excluded because their feces could not be collected, e.g., they left the hospital before sample collection. The subjects consisted of 11 males and 10 females ranging from 10 to 104 days of age, with an average age of 35.3 days. The average weight was 2,458 g, ranging from 1,186 to 4,545 g. Of the 21 subjects, 18 acquired MRSA during their stay in the hospitals and three were positive on admission. No subjects had gastrointestinal symptoms, such as diarrhea or vomiting.

### Isolation of MRSA strains

Nasal strains were collected using SEEDSWAB No. 2 (Eiken chemical Co. Ltd, Tokyo, Japan) and samples were inoculated onto two separate mannitol salt agar plates, one containing 2 mg/L oxacillin and one 10 mg/L cefoxitin. For fecal samples, 50 mg of stool was diluted with saline to 5% w/v, then was further diluted 10^2^-fold and 10^4^-fold. A 100 μl portion of each diluted sample was inoculated on two separate mannitol-salt agars containing 2 mg/L of oxacillin and 10 mg/L of cefoxitin. The number of MRSA strains in each fecal sample was estimated by counting the number of yellow-colored colonies grown on the selective medium. The yellow-colored colonies grown on the plates after incubation at 37 degrees Celsius for 48 hours were regarded to be MRSA strains, which were later confirmed using PCR of *mecA* and *femA*.

### Validation and characterization of MRSA strains

We extracted chromosomal DNA from 1–4 yellow-colored colonies on the agar plates using Cica Geneous**®** DNA Extraction Reagent (Kanto chemical Co., Tokyo, Japan) and conducted multiplex PCR using the Cica Geneus**®** Staph POT KIT (Kanto chemical Co., Tokyo, Japan). The kit contained 23 primer pairs identifying *femA* as a marker of *S. aureus*, five genes related to Staphylococcal cassette chromosome *mec* (SCC*mec*) elements, two genes located on the *S. aureus* chromosome and 15 genes located on mobile genetic elements, e.g., bacteriophages. The strains were regarded to be MRSA when both *mecA* and *femA* were identified.

### Molecular characterization of MRSA

Based on the results of multiplex PCRs with Staph POT kit, representative isolates from the nasal swabs and feces were chosen and characterized according to the multilocus sequence type (MLST), *spa* type, SCC*mec* type and presence of exotoxin genes. Chromosomal DNA was extracted using the DNeasy Tissue Kit (Qiagen, Valencia, USA). The SCC*mec* elements were identified using multiplex PCRs, as described by Kondo et al. [14]. The subtype of each SCC*mec* type was determined using PCR with primer pairs, as previously described. MLST and *spa*-type were determined as previously described [15,16]. Carriage of exotoxin genes, including *eta* and *etb* for exfoliative toxins a and b, *lukS-PV* and *lukF-PV* for Panton-Valentine Leukocidin and *tst* for Toxic shock syndrome toxin-1 (*tst*), was detected using PCR, as previously described [17].

### Ethical approval

This study was approved by the Ethical Committee of two participating hospitals and the written informed consent was obtained from the person in parental authority for the collection of samples and the publication of the analysed results.

## Results

### Isolation of MRSA strains from feces and nasal swabs

The feces of 21 MRSA screening-positive patients was diluted and inoculated on two separate mannitol-salt agar plates containing oxacillin or cefoxitin. Many yellow-colored colonies grew on the agar plates in all patients (Table 1). The number of yellow-colored colonies ranged from 4.0 × 10^2^ to 2.8 × 10^8^ of colony forming units (CFU)/g feces, with 1.7 × 10^7^ CFU/g feces on average. At the same time, the nasal swabs of 21 patients were streaked onto two separate mannitol-salt agar plates containing oxacillin or cefoxitin. Many yellow-colored colonies grew on the plates in all patients. One to four yellow-colored isolates from the nasal and fecal samples were chosen at random and subjected to multiple PCR with the Staph POT KIT. All tested strains were *mecA-* and *femA-* positive and therefore classified as MRSA. The data indicated that the feces of the patients with nasal colonization contained MRSA strains at significant amounts, although the numbers of MRSA colonies varied.

**Table 1:** Isolation of MRSA strains from the nasal swabs and feces of the patients in the NICU

**Patients**	**Growth on agar plates with antibiotics**				**Numbers of yellowish colony isolated from stool sample**^**a **^**(CFU/g)**
	**Nasal swabs**		**Feces**		
	**OXA**	**CFX**	**OXA**	**CFX**	
A	+	+	+	-	4.0 × 10^6^
B	+	+	+	+	1.6 × 10^5^
C	+	+	+	+	2.9 × 10^6^
D	+	+	+	+	3.1 × 10^6^
E	-	+	+	+	1.6 × 10^3^
F	+	+	+	+	2.4 × 10^6^
G	+	+	+	+	2.4 × 10^7^
H	+	+	+	+	4.7 × 10^6^
I	+	+	+	-	6.0 × 10^4^
J	+	+	+	-	1.6 × 10^3^
K	+	+	+	-	1.1 × 10^7^
L	+	NT	+	NT	6.0 × 10^6^
M	+	+	+	-	1.6 × 10^5^
N	+	+	+	+	2.9 × 10^6^
O	+	+	+	+	2.5 × 10^6^
P	+	+	+	+	2.8 × 10^8^
Q	+	+	+	+	4.0 × 10^2^
R	+	+	+	+	1.6 × 10^6^
S	+	+	+	+	1.0 × 10^3^
T	+	+	+	+	2.7 × 10^6^
U	+	+	+	+	2.9 × 10^6^

*Abbreviations*: *OXA* Oxacillin, *CFX* Cefoxitin, *CFU* colony forming units, *NT* not tested.

^a^Average number of yellow-colored colonies grown on agar plates with oxacillin.

### Comparisons of the carriage of ORFs by fecal and nasal strains

**Figure 1:**
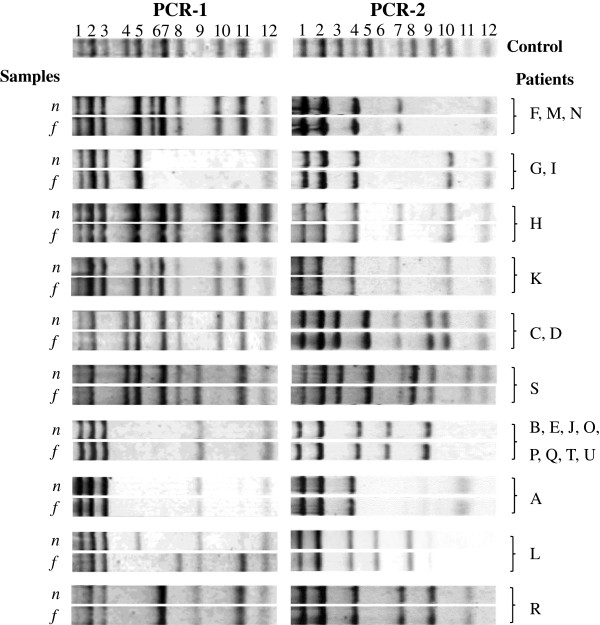
**Comparisons of amplified DNA fragments with two multiplex PCRs.** Abbreviations: *n*, isolates from nasal swabs; *f*, isolates from feces. For PCR-1: 1, *femA*; 2, *mecA*; 3, IS*1272*; 4, *kdpC*; 5, *tnpB* in Tn*554*; 6, ORF SV0850 in phi mu1; 7, ORF SAV0898 in phi mu1; 8, ORF SAV0866 in phi mu1; 9, ORF SA1774 in phi N315; 10, ORF SAV1974 in phi Sa 3mu; 11, ORF SAV0855 in phi mu1; 12, Genomic Island SaGIm. For PCR-2: 1, *femA*; 2, type 2 *ccrA*; 3, SA2259; 4, MW0919; 5, *mecI*; 6, ORF SAV0881 in phi mu1; 7, ORF 175 in phi SLT; 8, ORF SA1801 in phi N315; 9, ORF SAV0913 in phi mu1; 10, ORF 182 in phi SLT; 11, ORF SAV1998 in phi Sa 3mu; 12, ORF 2 in phi PV83.

To compare MRSA strains isolated from feces and nasal swabs of each patient, we firstly conducted two multiplex PCRs with Staph POT KIT by choosing 1–4 strains obtained from the feces and nasal swabs of the 21 patients. Two multiplex PCRs could amplify DNA fragments with related to SCC*mec* elements, two open reading frames (ORF) on the chromosome and 15 ORFs on the mobile genetic elements, e.g., lysogenized bacteriophages as listed in Table 2. Representative banding patterns of amplified DNA fragments from nasal and fecal strains isolated from the same patient are shown in Figure 1. In 17 of the 21 patients, exactly the same size and number of DNA fragments were generated using DNA samples from the feces and nasal swabs. In three patients (G, H and K), the dominant strains were identical, but other strains exhibiting different amplification patterns with ORFs in lysogenized bacteriophages were also identified: a nasal isolate of patient G, a fecal isolate of patient H and a nasal isolate of patient K. In a patient (L), different ORFs related to lysogenized bacteriophages were generated, although the amplified DNA fragments in association with the five ORFs in the SCC*mec* elements and two ORFs on the chromosome were the same.

**Table 2:** Characterization of the isolates using multiple PCR

**Patients**	**Samples**	**Number of tested strains**	**Numbers of amplified DNA fragments**^**a **^**with relate to**					
			**ORFs**^**b **^**on SCC** ***mec *** **elements**		**ORFs**^**c **^**on chromosome**		**ORFs**^**d **^**on mobile genetic elements**	
A	Nasal swab	4	IS*1272* / type 2 *ccr A*	4 / 4	MW0919	4 / 4	SA1774 / SaGlm / SAV1998	4 / 4
	Feces	2	IS*1272* / type 2 *ccr A*	2 / 2	MW0919	2 / 2	SA1774 / SaGlm / SAV1998	2 / 2
B	Nasal swab	4	IS*1272* / type 2 *ccr A*	4 / 4	MW0919	4 / 4	SA1774 / SaGlm / SAV0881 / SA1801	4 / 4
	Feces	4	IS*1272* / type 2 *ccr A*	4 / 4	MW0919	4 / 4	SA1774 / SaGlm / SAV0881 / SA1801	4 / 4
C	Nasal swab	4	*mecI* / type 2 *ccrA* /*kdpC*	4 / 4	SA2259	4 / 4	*tnp*B / SAV0898 / SAV0866 / SAV1974 / SAV0855 / SaGlm / SLTorf175 / SAV0913 / SLTorf182 / PV83orf2	4 / 4
	Feces	4	*mecI* / type 2 *ccrA* /*kdpC*	4 / 4	SA2259	4 / 4	*tnp*B / SAV0898 / SAV0866 / SAV1974 / SAV0855 / SaGlm / SLTorf175 / SAV0913 / SLTorf182 / PV83orf2	4 / 4
D	Nasal swab	4	*mecI* / type 2 *ccrA* /*kdpC*	4 / 4	SA2259	4 / 4	*tnp*B / SAV0898 / SAV0866 / SAV1974 / SAV0855 / SaGlm / SLTorf175 / SAV0913 / SLTorf182 / PV83orf2	4 / 4
	Feces	4	*mecI* / type 2 *ccrA* /*kdpC*	4 / 4	SA2259	4 / 4	*tnp*B / SAV0898 / SAV0866 / SAV1974 / SAV0855 / SaGlm / SLTorf175 / SAV0913 / SLTorf182 / PV83orf2	4 / 4
E	Nasal swab	2	IS*1272* / type 2 *ccr A*	2 / 2	MW0919	2 / 2	SA1774 / SaGlm / SAV0881 / SA1801	2 / 2
	Feces	4	IS*1272* / type 2 *ccr A*	4 / 4	MW0919	4 / 4	SA1774 / SaGlm / SAV0881 / SA1801	4 / 4
F	Nasal swab	4	IS*1272* / type 2 *ccr A*	4 / 4	MW0919	4 / 4	*tnp*B / SAV0850 / SAV0898 / SAV0866 / SAV1974 / SAV0855 / SaGlm / SLTorf175 / PV83orf2	4 / 4
	Feces	4	IS*1272* / type 2 *ccr A*	4 / 4	MW0919	4 / 4	*tnp*B / SAV0850 / SAV0898 / SAV0866 / SAV1974 / SAV0855 / SaGlm / SLTorf175 / PV83orf2	4 / 4
G	Nasal swab	3	IS*1272* / type 2 *ccr A*	3 / 3	MW0919	3 / 3	*tnp*B / SaGlm / SLTorf182 / PV83orf2	2 / 3
							*tnp*B / SAV0850 / SAV0898 / SAV0866 / SAV1974 / SAV0855 / SaGlm / SLTorf175 / PV83orf2	1 / 3
	Feces	4	IS*1272* / type 2 *ccr A*	4 / 4	MW0919	4 / 4	*tnp*B / SaGlm / SLTorf182 / PV83orf2	4 / 4
H	Nasal swab	4	IS*1272* / type 2 *ccr A*	4 / 4	MW0919	4 / 4	*tnp*B / SAV0898 / SAV0866 / SAV1974 / SAV0855 / SaGlm / SLTorf175 / SLTorf182 / PV83orf2	4 / 4
	Feces	4	IS*1272* / type 2 *ccr A*	4 / 4	MW0919	4 / 4	*tnp*B / SAV0898 / SAV0866 / SAV1974 / SAV0855 / SaGlm / SLTorf175 / SLTorf182 / PV83orf2	2 / 4
							*tnp*B / SAV0850 / SAV0898 / SAV0866 / SAV1974 / SAV0855 / SaGlm / SLTorf175 / SLTorf182 / PV83orf2	2 / 4
I	Nasal swab	4	IS*1272* / type 2 *ccr A*	4 / 4	MW0919	4 / 4	*tnp*B / SaGlm / SLTorf182 / PV83orf2	4 / 4
	Feces	2	IS*1272* / type 2 *ccr A*	2 / 2	MW0919	2 / 2	*tnp*B / SaGlm / SLTorf182 / PV83orf2	2 / 2
J	Nasal swab	4	IS*1272* / type 2 *ccr A*	4 / 4	MW0919	4 / 4	SA1774 / SaGlm / SAV0881 / SA1801	4 / 4
	Feces	2	IS*1272* / type 2 *ccr A*	2 / 2	MW0919	2 / 2	SA1774 / SaGlm / SAV0881 / SA1801	2 / 2
K	Nasal swab	4	IS*1272* / type 2 *ccr A*	4 / 4	MW0919	4 / 4	*tnp*B / SAV0850 / SAV0898 / SAV0866 / SAV1974 / SAV0855 / SaGlm / SLTorf175 / SLTorf182 / PV83orf2	3 / 4
							*tnp*B / SAV0898 / SAV0866 / SAV1974 / SAV0855 / SaGlm / SLTorf175 / SLTorf182 / PV83orf2	1 / 4
	Feces	2	IS*1272* / type 2 *ccr A*	2 / 2	MW0919	2 / 2	*tnp*B / SAV0850 / SAV0898 / SAV0866 / SAV1974 / SAV0855 / SaGlm / SLTorf175 / SLTorf182 / PV83orf2	2 / 2
L	Nasal swab	1	IS*1272* / type 2 *ccr A*	1 / 1	MW0919	1 / 1	*tnp*B/ SAV1774 / SaGlm / SAV0881 / SAV1801	1 / 1
	Feces	1	IS*1272* / type 2 *ccr A*	1 / 1	MW0919	1 / 1	SAV0886 / SA1774 / SAV0885 / SaGlm / SAV0881 / SAV1801	1 / 1
M	Nasal swab	4	IS*1272* / type 2 *ccr A*	4 / 4	MW0919	4 / 4	*tnp*B / SAV0850 / SAV0898 / SAV0866 / SAV1974 / SAV0855 / SaGlm / SLTorf175 / PV83orf2	4 / 4
	Feces	2	IS*1272* / type 2 *ccr A*	2 / 2	MW0919	2 / 2	*tnp*B / SAV0850 / SAV0898 / SAV0866 / SAV1974 / SAV0855 / SaGlm / SLTorf175 / PV83orf2	2 / 2
N	Nasal swab	4	IS*1272* / type 2 *ccr A*	4 / 4	MW0919	4 / 4	*tnp*B / SAV0850 / SAV0898 / SAV0866 / SAV1974 / SAV0855 / SaGlm / SLTorf175 / PV83orf2	4 / 4
	Feces	4	IS*1272* / type 2 *ccr A*	4 / 4	MW0919	4 / 4	*tnp*B / SAV0850 / SAV0898 / SAV0866 / SAV1974 / SAV0855 / SaGlm / SLTorf175 / PV83orf2	4 / 4
O	Nasal swab	4	IS*1272* / type 2 *ccr A*	4 / 4	MW0919	4 / 4	SA1774 / SaGlm / SAV0881 / SA1801	4 / 4
	Feces	4	IS*1272* / type 2 *ccr A*	4 / 4	MW0919	4 / 4	SA1774 / SaGlm / SAV0881 / SA1801	4 / 4
P	Nasal swab	4	IS*1272* / type 2 *ccr A*	4 / 4	MW0919	4 / 4	SA1774 / SaGlm / SAV0881 / SA1801	4 / 4
	Feces	4	IS*1272* / type 2 *ccr A*	4 / 4	MW0919	4 / 4	SA1774 / SaGlm / SAV0881 / SA1801	4 / 4
Q	Nasal swab	4	IS*1272* / type 2 *ccr A*	4 / 4	MW0919	4 / 4	SA1774 / SaGlm / SAV0881 / SA1801	4 / 4
	Feces	4	IS*1272* / type 2 *ccr A*	4 / 4	MW0919	4 / 4	SA1774 / SaGlm / SAV0881 / SA1801	4 / 4
R	Nasal swab	4	IS*1272* / type 2 *ccr A*	4 / 4	MW0919	4 / 4	SAV0898 / SAV1774 / SAV0855 / SaGlm / SLTorf175 / SAV0913 / SAV1998	4 / 4
	Feces	4	IS*1272* / type 2 *ccr A*	4 / 4	MW0919	4 / 4	SAV0898 / SAV1774 / SAV0855 / SaGlm / SLTorf175 / SAV0913 / SAV1998	4 / 4
S	Nasal swab	4	*mecI* / type 2 *ccrA* /*kdp*C	4 / 4	SA2259	4 / 4	*tnp*B / SAV0898 / SAV0866 / SAV1774 / SAV0855 / SaGlm / SLTorf175 / SAV1801 / SAV0913 / SAV1998 / PV83orf2	4 / 4
	Feces	4	*mecI* / type 2 *ccrA* /*kdp*C	4 / 4	SA2259	4 / 4	*tnp*B / SAV0898 / SAV0866 / SAV1774 / SAV0855 / SaGlm / SLTorf175 / SAV1801 / SAV0913 / SAV1998 / PV83orf2	4 / 4
T	Nasal swab	4	IS*1272* / type 2 *ccr A*	4 / 4	MW0919	4 / 4	SA1774 / SaGlm / SAV0881 / SA1801	4 / 4
	Feces	4	IS*1272* / type 2 *ccr A*	4 / 4	MW0919	4 / 4	SA1774 / SaGlm / SAV0881 / SA1801	4 / 4
U	Nasal swab	4	IS*1272* / type 2 *ccr A*	4 / 4	MW0919	4 / 4	SA1774 / SaGlm / SAV0881 / SA1801	4 / 4
	Feces	4	IS*1272* / type 2 *ccr A*	4 / 4	MW0919	4 / 4	SA1774 / SaGlm / SAV0881 / SA1801	4 / 4

*Abbreviations*: *ORFs* open reading frames.

^a^The ORFs amplified by two multiplex PCR using the Cica Geneus Staph POT KIT are listed.

^b^PCR-positive ORFs related to SCCmec elements. The results of PCR of five genes, mecA, mecI, IS1272, type 2 ccrA, and kdpC located in type IIa SCCmec, are listed. PCR-positive genes, except for mecA, are listed.

^c^PCR-positive ORFs located on the chromosomes of N315(SA2259) and MW2(MW0919).

^d^PCR-positive ORFs related to mobile genetic elements are listed. Fifteen ORFs were targeted for PCR, as follows: *tnp*B in the transposon Tn*554*, a ORF on the genome islands SaGIm, and 12 ORFs on the lysogenized bacteriophages (SA1774 and SA1801 for phi N315; SAV0881, SAV0850, SAV0855, SAV0866, SAV0881, SAV0898 and SAV0913 for phi Mu50A; SAV1974 and SAV1998 for phi Mu50B; orf 175 and orf 182 for phi SLT; and orf2 for phi PV83).

### Molecular characterization of the MRSA strains

The MLST genotypes, SCC*mec* types and *spa* types were determined in one representative isolate from each feces sample among the 21 patients. Three clonal complexes (CC1, CC5 and CC8), four SCC*mec* types (IIa, IVa, IVb and IVl), and six *spa* types (2, 59, 606, 855, 1499 and 1500) were identified. Consequently, 21 strains were classified into four clones (Table 3). CC8-SCC*mec* IVl was the most prominent clone (10 of 21), followed by CC1-SCC*mec* IVa (7 of 21), CC5-SCC*mec* IIa (3 of 21) and CC8-SCC*mec* IVb (1 of 21). The carriage of the four exotoxin genes was examined. Eleven of the 21 strains carried the *tst* gene, including 10 CC8-SCC*mec* IVl strains and one CC5-SCC*mec* IIa strain. In contrast, the *eta, etb* an *lukS,F*-PV genes were not identified in any of the tested strains. Next, we chose one representative isolate from each nasal swab among the 21 patients and examined the SCC*mec* types, *spa*-types and exotoxin repertoire. The characteristics of the nasal strains were exactly identical to those of the fecal strains. Taken together with the results of multiplex PCR shown in Table 2, the feces of the nasal carriers of MRSA contained the same MRSA clones as the strain isolated from the nasal swabs. Three MRSA clones, CC5-SCC*mec* IIa, CC8-SCC*mec* IVl and CC8-SCC*mec* IVb, were identified at the *J* hospital and one clone, CC1-SCC*mec* IVa, was identified at the *S* hospital.

**Table 3:** Molecular characterization of the MRSA strains isolated from the feces and nasal swabs

**Strains**^**a**^	**MLST**		***spa *** **types**	**SCC** ***mec *** **types**	**Exotoxin genes**				**Isolated at**^**b**^
	**ST**	**CC**			***tst***	***eta***	***etb***	***lukS, F-PV***	
A*f*	8	8	1499	IVl	+	-	-	-	*J*
A*n*	NT	NT	1499	IVl	+	-	-	-	
B*f*	8	8	606	IVl	+	-	-	-	*J*
B*n*	NT	NT	606	IVl	+	-	-	-	
C*f*	764	5	2	IIa	-	-	-	-	*J*^*c*^
C*n*	NT	NT	2	IIa	-	-	-	-	
D*f*	764	5	2	IIa	-	-	-	-	*J*^*c*^
D*n*	NT	NT	2	IIa	-	-	-	-	
E*f*	8	8	606	IVl	+	-	-	-	*J*
E*n*	NT	NT	606	IVl	+	-	-	-	
F*f*	2763	1	1500	IVa	-	-	-	-	*S*
F*n*	NT	NT	1500	IVa	-	-	-	-	
G*f*	2764	1	855	IVa	-	-	-	-	*S*
G*n*	NT	NT	855	IVa	-	-	-	-	
H*f*	2764	1	855	IVa	-	-	-	-	*S*
H*n*	NT	NT	855	IVa	-	-	-	-	
I*f*	1	1	855	IVa	-	-	-	-	*S*
I*n*	NT	NT	855	IVa	-	-	-	-	
J*f*	8	8	606	IVl	+	-	-	-	*J*
J*n*	NT	NT	606	IVl	+	-	-	-	
K*f*	2763	1	855	IVa	-	-	-	-	*S*
K*n*	NT	NT	855	IVa	-	-	-	-	
L*f*	8	8	59	IVb	-	-	-	-	*J*
L*n*	NT	NT	59	IVb	-	-	-	-	
M*f*	2763	1	855	IVa	-	-	-	-	*S*
M*n*	NT	NT	855	IVa	-	-	-	-	
N*f*	2763	1	855	IVa	-	-	-	-	*S*
N*n*	NT	NT	855	IVa	-	-	-	-	
O*f*	8	8	606	IVl	+	-	-	-	*J*
O*n*	NT	NT	606	IVl	+	-	-	-	
P*f*	8	8	606	IVl	+	-	-	-	*J*
P*n*	NT	NT	606	IVl	+	-	-	-	
Q*f*	8	8	606	IVl	+	-	-	-	*J*
Q*n*	NT	NT	606	IVl	+	-	-	-	
R*f*	8	8	606	IVl	+	-	-	-	*J*
R*n*	NT	NT	606	IVl	+	-	-	-	
S*f*	5	5	2	IIa	+	-	-	-	*J*^*c*^
S*n*	NT	NT	2	IIa	+	-	-	-	
T*f*	8	8	606	IVl	+	-	-	-	*J*
T*n*	NT	NT	606	IVl	+	-	-	-	
U*f*	8	8	606	IVl	+	-	-	-	*J*
U*n*	NT	NT	606	IVl	+	-	-	-	

*Abbreviations*: *f* feces, *n* nasal swabs, *NT* not tested.

^a^Strains isolated from feces and nasal swabs of patients A-U.

^b^Strains isolated at two hospitals: Juntendo University Hospital (*J*) and Juntendo University Shizuoka Hospital (*S*).

^c^Patients C, D and S had already been colonized with MRSA before being transferred to hospital J from previous facilities.

## Discussion

### The feces of the infants contained significant amount of MRSA pathogens

In this study, we found that the feces of all 21 patients, from whom nasal cavity MRSA strains were isolated, contained MRSA. Adlerberth I. et al. reported the rates of *S. aureus* isolation from feces were 40% to 80% in healthy newborns and infants ranging from 7 days to 1 year of age [18,19]. Acton et al. reported a detection rate of intestinal carriage in healthy individuals and patients of 20% for *S. aureus* and 9% for MRSA, which is approximately half of that observed for nasal carriage [10]. Here we showed that infant-nasal MRSA carriers consistently evacuate a lot of cells of MRSA in their feces. Many reports advocate establishing better screening methods for identifying MRSA carriers by evaluating optimum surveillance sites, e.g., the nasal cavity, skin, feces and/or rectum, in order to isolate pathogens [8,9]. To screen for MRSA as part of an active surveillance program, nasal swabs are usually used because the method is easy to perform and has higher sensitivity than other methods, while other sites have been used for complementation [2,7,20].

A lot of cells of MRSA were isolated from the feces of infants. The amount was greater than those reported in adults, similar to the previous report [21]. The intestinal flora of adults is generally occupied by established microflora, which may help to prevent the colonization of newly incorporated bacteria, known as the phenomenon of “colonization resistance” [22]. In contrast, the intestinal flora of neonates and infants, especially premature babies admitted to the NICU, have not yet been established. Therefore, we presume that MRSA strains are able to propagate in or colonize the intestinal tract of infants more efficiently than that of adults.

### The feces of nasal MRSA carriers is associated with a high risk for the dissemination of MRSA

In hospitals, the horizontal transmission of infective substances from an infected patient to another patient via contact with medical staff is likely to occur, as previously described. However, transmission via direct contact can be controlled by thoroughly implementing standard precautions, regardless of the presence of MRSA colonization. Furthermore, it is widely recognized that the stool and vomit excreted from gastroenteritis patients contain many pathogens, e.g., rotavirus, which can cause a secondary infection [23-25]. We suggest that healthcare workers should recognize the feces from nasal MRSA carriers as a potential source of MRSA strains that cause transmission, although gastrointestinal symptoms may not occur in all patients with fecal MRSA colonization [26]. Feces containing MRSA may serve as a source of contamination due to the possibility of spreading to surrounding surfaces by contact with healthcare workers’ hands. Our data suggested that more careful attention should be paid for the handling of excrement in the case of newborn babies or infants than that of adults. When changing diapers or cloths and bathing neonates and infants in the NICU, healthcare workers may come in contact with MRSA. In the cases of treatment failure, e.g., inappropriate precautions, hand washing or hand hygiene using antiseptic agents, the transmission of MRSA is likely to occur. Although there are several studies regarding the intestinal carriage of *S. aureus* including MRSA, there were few studies that examined molecular epidemiological characteristics of MRSA strains from two sources at the same time. In this study, the MRSA clones contained in the feces were identical to those isolated from the nasal cavity, suggesting that MRSA strains in the nasal cavity are carried into the intestines, and the feces of MRSA nasal carriers should be regarded as a source of transmission of MRSA. Since the number of MRSA colonies in the feces was greater than that observed in the nasal cavity, the risk of transmission is higher in cases involving contact with feces than with nasal secretions. However, the subjects of this study were chosen from the patients who admitted to NICU. Threfore, it is unclear whether the same results were obtained in the cases of healthy infants or adult patients. Further research need to be conducted to clarify these questions.

### Characteristics MRSA clones isolated from feces and nasal swabs

To know identities of MRSA clones contained in the feces and nasal swabs, we firstly screened MRSA strains in plural with multiplex PCRs that can identify ORFs in SCC*mec* elements, some chromosomal genes of CC1 and CC5 strains, and genes in mobile genetic elements, e.g., bacteriophage. We did not regard the method as a one to be used instead of pulsed field gel electrophresis or to be used to determine MRSA clone. Here we used the PCRs as a compendium method to examine the identities of the carriage of tested ORFs between several strains. A majority of strain isolated from feces and nasal swabs of every one person were the same, suggesting that the same clone might be isolated. These data indicated that MRSA strains at the nasal cavity entered to digestive tract, and propagated there.

Further detailed investigation revealed characteristic of MRSA strains of two hospitals. At the *J* hospital, the CC8-SCC*mec* IVl clone was predominant, although two other clones, CC5-SCC*mec* IIa and CC8-SCC*mec* IVb, were also identified. At the *S* hospital, only the CC1-SCC*mec* IVa clone was identified. Of the 21 carriers, three were positive on admission to the units and 18 acquired MRSA during their stay in hospitals. Three CC5-SCC*mec* IIa strains were identified in the feces of patients transferred from other facilities who carried MRSA before hospitalization in the *J* hospital. These data suggest that 18 patients acquired the specific MRSA strains disseminated at each hospital. Type II SCC*mec* strains are still dominant in Japanese hospitals, and there are reports that type IV SCC*mec* strains are disseminated in the Japanese community [27]. Yanagihara et al. reported that SCC*mec* IV strains are predominantly distributed in the outpatient clinics [28]. It is curious that the MRSA clones disseminated in the NICU were not identical to dominant MRSA clone disseminating in other wards, but rather were similar to those isolated from the outpatient departments. It is well known that the epidemiological distribution of MRSA strains varies according to region and age [29-32]. MRSA strains are classified into healthcare-associated (HA-MRSA) and community-associated (CA-MRSA) strains [33,34]. Recent studies have shown that MRSA strains identified in the community showed characteristics that were distinct from the HA-MRSA, and such CA-MRSA clones are sometimes isolated from hospitals [30].

Recently, Iwao et al. reported that CC8-SCC*mec* IVl strains that carry the toxic shock syndrome toxin gene have been detected in the Japanese community [35]. This finding is confirmed by our observation that the CC8-SCC*mec* IVl strain is the dominant strain isolated from outpatient clinics of dermatology (Hosoya et al. unpublished data). In the current study, SCC*mec* IV strains were dominantly isolated from patients admitted to our hospital at birth. These data suggest that MRSA clones emerging in the community are transmitted into and disseminated throughout the hospital. Toxic shock syndrome toxin is a well-known super antigen that causes neonatal toxic shock syndrome-like exanthematous disease in neonates and infants [36]. All *tst*-positive isolates produce a considerable amount of toxic shock syndrome toxins. Fortunately, most patients did not experience severe symptoms, although the possibility of life-threatening syndrome remains. The root of transmission for the transfer of MRSA clones originating in the Japanese community into the NICU remains unknown; however, one possibility is that the pathogen is brought into the hospital by the patient’s mother or other family members. Studies investigating the transmission of MRSA clones colonized in the nasal cavity in family members are required to clarify this issue.

## Conclusions

The feces of the investigated MRSA carriers contained the same MRSA clones obtained from the nasal swabs in considerable amounts, suggesting that the feces of MRSA carriers is associated with a high risk for disseminating MRSA.

## Abbreviations

MRSA: Methicillin-resistant *Staphylococcus aureus*; NICU: Neonatal intensive care unit; MLST: Multilocus sequence typing; SCC: Staphylococcal cassette chromosome; CC: Clonal complex; CFU: Colony forming unit; ORF: Open reading frame; HA-MRSA: Healthcare-associated methicillin-resistant *Staphylococcus aureus*; CA-MRSA: Community-associated methicillin-resistant *Staphylococcus aureus.*

## Competing interests

The authors declare that they have no any competing interests.

## Authors’ contributions

AN and TI designed the research and prepared manuscript. AN, AT, MN, MK, and KH planned infection control at NICU and collected nasal swabs and feces at the NICU. AN isolated strains from the samples. AN, KH, and YL characterized strains. KH and TS supervised this study giving suggestions for the research and for the draft of the manuscript. All authors read and approved the final manuscript.
